# Smad7:β-catenin complex regulates myogenic gene transcription

**DOI:** 10.1038/s41419-019-1615-0

**Published:** 2019-05-16

**Authors:** Soma Tripathi, Tetsuaki Miyake, John C. McDermott

**Affiliations:** 1 Department of Biology, York University, Toronto, ON M3J 1P3 Canada; 2 Muscle Health Research Centre (MHRC), York University, Toronto, ON M3J 1P3 Canada; 3 Centre for Research in Biomolecular Interactions (CRBI), York University, Toronto, ON M3J 1P3 Canada; 4 Centre for Research in Mass Spectrometry (CRMS), York University, Toronto, ON M3J 1P3 Canada

**Keywords:** DNA, Transcriptional regulatory elements, Stem-cell research

## Abstract

Recent reports indicate that Smad7 promotes skeletal muscle differentiation and growth. We previously documented a non-canonical role of nuclear Smad7 during myogenesis, independent of its role in TGF-β signaling. Here further characterization of the myogenic function of Smad7 revealed β-catenin as a Smad7 interacting protein. Biochemical analysis identified a Smad7 interaction domain (SID) between aa575 and aa683 of β-catenin. Reporter gene analysis and chromatin immunoprecipitation demonstrated that Smad7 and β-catenin are cooperatively recruited to the extensively characterized *ckm* promoter proximal region to facilitate its muscle restricted transcriptional activation in myogenic cells. Depletion of endogenous Smad7 and β-catenin in muscle cells reduced *ckm* promoter activity indicating their role during myogenesis. Deletion of the β-catenin SID substantially reduced the effect of Smad7 on the *ckm* promoter and exogenous expression of SID abolished β-catenin function, indicating that SID functions as a *trans* dominant-negative regulator of β-catenin activity. β-catenin interaction with the Mediator kinase complex through its Med12 subunit led us to identify MED13 as an additional Smad7-binding partner. Collectively, these studies document a novel function of a Smad7-MED12/13-β-catenin complex at the *ckm* locus, indicating a key role of this complex in the program of myogenic gene expression underlying skeletal muscle development and regeneration.

## Introduction

Developmental myogenesis, the process of terminal differentiation of skeletal muscle progenitor cells, consists of a series of well-characterized highly regulated steps that has become a paradigm for lineage acquisition and cellular differentiation^1–5^. During embryogenesis, pluripotent mesodermal stem cells commit to become myogenic progenitor cells^6^. Commitment to the myogenic lineage results in a binary state of either maintenance of proliferative potential and multipotency or, on appropriate cues, withdrawal from the cell cycle, activation of a battery of structural, contractile, and metabolic genes and ultimately formation of multi-nucleated, electrically excitable myofibers^1,7,8^. Also, in adult skeletal muscle, resident stem cells (satellite cells) located between the basal lamina and the plasma membrane of mature muscle fibers^9^ recapitulate this “myogenic program” of differentiation in response to injury or for routine maintenance of the muscle tissue^10^.

How networks of transcriptional regulators exert molecular control over developmental and adult myogenesis has been a prevalent theme in understanding the molecular control of ontogeny, physiology, and pathology of the striated muscle^10–12^. Extensive biochemical and genetic evidence has implicated a family of DNA-binding transcriptional regulatory proteins encoded by the myogenic regulatory factor (MRF) genes, *myf5*, *myod1*, *myogenin* (*myog*), and *mrf4*, in myogenesis^10,13^. In conjunction with the proteins encoded by the *myocyte enhancer factor 2* (*mef2a-d*) gene family, the MRF’s activate an evolutionarily conserved program of gene expression, which leads to the generation of terminally differentiated skeletal muscle cells^14–16^. Understanding the *trans*-acting factors contributing to this process has been aided in tandem by extensive analysis of the *cis*-regulatory elements of muscle-restricted, differentiation-induced genes such as the *muscle creatine kinase* (*ckm*) gene^17–19^.

In addition to the central role played by the MRF/MEF2 axis in myogenesis^20^, other transcription factors have been implicated in the control of myogenic differentiation such as Six 1 and 4^21–23^, AP-1^24,25^, β-catenin^26–28^, and Smad7^29–31^. Since our initial observations of the pro-myogenic role of Smad7 in cultured muscle cells^29^, we have documented a novel nuclear role for Smad7 in muscle that is essentially independent of its “canonical” role as a repressor of transforming growth factor (TGF)-β signaling^31^. In addition, other groups have documented an in vivo role for Smad7 in the skeletal muscle^30^. Understanding the nature of the ancillary role played by Smad7 and other transcriptional regulators at muscle-restricted genes is therefore of some importance for our overall understanding of the molecular programming of myogenic identity.

In view of the “non-canonical” nuclear role of Smad7 alluded to above, the mechanistic basis by which it contributes to the myogenic differentiation program is so far incompletely understood. We were intrigued by a report identifying a protein–protein interaction (PPI) between Smad7 and β-catenin in human prostate cancer cells^32^ since both are known, independently, to be key regulators of muscle gene expression in a variety of contexts. We therefore assessed this putative PPI in cultured muscle cells. Our data support a robust interaction between Smad7 and β-catenin that contributes to the transcriptional control of a key myogenic promoter (*ckm*). We further extended these observations in characterizing the recruitment of Mediator components (Med12/13) by the β-catenin/Smad7 complex, thus connecting the muscle transcriptosome assembled on enhancers such as *ckm* to the basal transcription machinery. Integration of the Smad7-β-catenin complex into the network of proteins regulating the “myogenic program” expands our understanding of the unique molecular wiring encoding myogenic differentiation, growth and repair.

## Materials and methods

### Cell culture

C2C12 myoblasts were obtained from the American Type Culture Collection. Cells were cultured in growth medium (GM) consisting of high-glucose Dulbecco’s modified Eagle’s medium (DMEM, Gibco), 10% fetal bovine serum (FBS), and L-Glutamine (HyClone) supplemented with 1% penicillin–streptomycin (Invitrogen, ThermoFisher). Myotube formation was induced by replacing GM with differentiation medium (DM), consisting of DMEM supplemented with 2% horse serum (Atlanta Biologicals) and 1% penicillin–streptomycin. Cells were maintained in an incubator at 95% humidity, 5% CO_2_, and 37 °C.

### Transfections

For ectopic protein expression, cells were transfected using the calcium phosphate precipitation method for transcription reporter assays. Cells were re-fed 16 h post-transfection and harvested. For small interfering RNA (siRNA) experiments, cells were transfected with Lipofectamine 2000 (Life Technologies) using instructions provided by the manufacturer and harvested 48 h later, unless otherwise indicated.

### Gene silencing

MISSION siRNA (Sigma-Aldrich) for rat and mouse *ctnnb1* siβ-catenin#1 (SASI_Rn01_00099925), siβ-catenin#2 (SASI_Rn01_00099923), siβ-catenin#3 (SASI_Rn01_00099924), siSmad7#1 (SASI_Mm02_00290887), siSmad7#2 (SASI_Mm02_00290886), siSmad7#3 (SASI_Mm02_00290885) and universal scrambled siRNA (SIC001) were used at 75 nM concentrations.

### Plasmids

Expression plasmids for Myc-His-tagged full-length Smad7, β-catenin-myc, transcription reporter assay constructs *pckm-luc* have been described previously^29,31,33^. β-catenin mutant expression plasmids were constructed by the ligation of PCR-amplified nucleotides corresponding to the indicated amino acid (aa) regions (aa575–683, aa1–574) at Hind III and Xho I sites of pcDNA3-EYFP or pcDNA3-3Xflag-8XHis, respectively. Constructs for expression of glutathione-S-transferase (GST)-fused β-catenin fragments were described previously^34^.

### Transcription reporter gene assays

Transcriptional reporter assays were performed using luciferase reporter plasmids along with expression constructs (indicated in the figure legends) and a *Renilla* plasmid (pRL-Renilla, Promega) as an internal control. Cells were washed with 1× phosphate-buffered saline and harvested in Luciferase Lysis Buffer (20 mM Tris pH 7.4 and 0.1% Triton X-100). Enzymatic activity was measured in each sample on a luminometer (Lumat LB, Berthold) using Luciferase assay substrate (E1501; Promega) or Renilla assay substrate (E2820; Promega). Luciferase activity values obtained were normalized to Renilla activity in the same cell extracts and expressed as fold activation to the control.

### Nuclear and cytoplasmic extraction

Nuclear and cytoplasmic extraction was obtained using the NE-PER Kit (78833; Thermo Scientific), as per the instructions provided by the manufacturer. Immunoblotting of extracellular signal-regulated kinase and c-Jun was used as the positive control for cytoplasmic and nuclear fractions, respectively.

### Western blot analysis

Total cellular protein extracts were prepared in NP-40 lysis buffer (0.5% (vol/vol)), 50 mM Tris-HCl (pH 8),150 mM NaCl, 10 mM sodium pyrophosphate, 1 mM EDTA (pH 8), and 0.1 M NaF supplemented with 1× protease inhibitor cocktail (P-8340; Sigma) and 0.5 mM sodium orthovanadate. Protein concentrations were determined by a standard Bradford assay. Equivalent amounts of protein were denatured in sodium dodecyl sulfate (SDS) loading buffer at 100 °C for 5 min and then run in SDS-polyacrylamide gels, followed by electrophoretic transfer to an Immobilon-FL polyvinylidene difluoride membrane (Millipore) as directed by the manufacturer. Blots were incubated with blocking buffer that consisted of 5% milk in Tris-buffered saline (TBS)-T (10 mM Tris-HCl, pH 8.0, 150 mM NaCl, 0.1% Tween 20) prior to the incubation with primary antibody at 4 °C overnight with gentle agitation. After three washes with TBS-T, appropriate horseradish peroxidase-conjugated secondary antibody (BioRad, 1:2000) were added for 2 h at room temperature. Protein/antibody immune-complexes were detected with Enhanced Chemiluminescence western blotting substrate (Pierce, ThermoFisher).

### Antibodies

Rabbit monoclonal for αSmad7 (ab124890) and polyclonal for αMED13 (ab76923) and αMED12 (ab70842) were purchased from Abcam. A rabbit polyclonal antibody was raised against GST-Smad7 according to the protocol approved by York University of Animal Care Committee. This was used for endogenous Smad7 immunoprecipitation (IP) and detection in cellular and nuclear extract. αβ-Catenin (pAb9562) and ChIP-grade αFlag antibody (mAb 14793S) were purchased from Cell signaling. Monoclonal αFlag antibody (F1804) was from Sigma. αMyc (9E10), αMyHC (MF20), and αMyogenin (F5D) were purchased from DSHB. αActin (Sc1616), αMyoD (sc304), and αCKM (sc-69878) were purchased from Santa Cruz.

### Co-immunoprecipitation (Co-IP)

For Co-IP, cells were harvested, and proteins were extracted as described above. IP was performed using an ImmunoCruz Optima Kit (Santa Cruz Biotechnology) according to the manufacturer’s instructions. Eluates were analyzed by western blotting as described above.

### Live-cell imaging

C2C12 cells were seeded onto the glass-bottom dishes (MatTek Corp). The cells were transfected for the expression of fluorescent-tagged proteins. Before imaging the cells, the media was replaced for FluoroBrite DMEM (Thermo Fisher Scientific) supplemented with 10% FBS. To mark nuclei, Hoechst 33342 (Sigma-Aldrich) was added to 2.5 µM into the media. After 30 min, the stained cells were visualized by a Carl Zeiss Spinning disc system (Zeiss Observer Z1 with Yokogawa CSU-X1 and AxioCam MRm camera) in the environment chamber (37 °C, 5% CO_2_). The raw images were processed by ZEN (Carl Zeiss) to obtain pseudo-colored micrographs.

### Chromatin immunoprecipitation (ChIP)

C2C12 myoblasts or Smad7-Flag ectopically expressing myoblasts were crosslinked with 1% formaldehyde followed by sonication to shear DNA strands attached to the protein. Smad7/β-catenin was immunoprecipitated with Flag or β-catenin antibody to obtain the protein–DNA complex. IP with IgG antibody served as controls. Immunoprecipitated DNA was reverse crosslinked, purified, and subjected to quantitative PCR (qPCR) using primers specific for the *ckm* promoter. Primers specific to *gapdh* was used as controls for *ckm* enrichment.

### GST pull down assay

For GST pull down assays, GST-Smad7 and 6XHis-β-catenin fusion proteins were produced in bacteria using standard protocol. Briefly, GST-Smad7- and 6XHis-β-catenin-expressing cells were sonicated and the proteins were purified with glutathione-agarose beads (Sigma-Aldrich G-4510). Protein concentrations were estimated by SDS-polyacrylamide gel electrophoresis and following Coomassie blue staining, using BSA for comparative estimation. GST-β-catenin fragments corresponding to aa (full length (FL), 1–100, 120–683, 422–683, 575–696, 575–683, 1–574) were utilized for mapping study. Five micrograms of GST FL Smad7, β-catenin (or the molar equivalent of the smaller GST, GST-β-catenin fragments), and 25 µl glutathione-agarose beads (50% slurry) were incubated in 600 µl NETN buffer (100 mM NaCl, 20 mM Tris-HCl (pH 8) 0.5 mM EDTA, 0.5% (vol/vol) NP-40) overnight at 4 °C. In experiments where GST-β-catenin was used to co-precipitate Smad7, GST-Smad7 was thrombin (GE Healthcare-27-0846-01) digested to yield the immune-complex without the GST tag (10 µl thrombin per mg fusion protein was incubated at room temperature for 17 h), and eluates were analyzed by western blotting.

## Results

### Smad7 and β-catenin are co-expressed and interact in myogenic cells

**Fig. 1:**
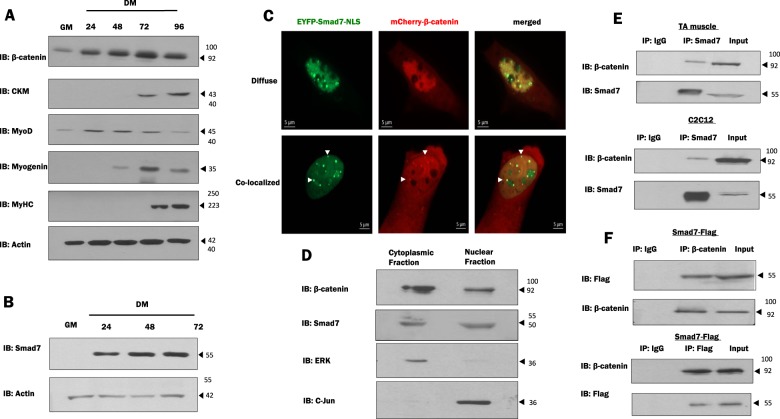
Smad7 and β-catenin interaction and expression during myogenic differentiation. **a** C2C12 myoblasts were cultured in growth media (GM) for 24 h, followed by differentiation media (DM) for designated times. Lysates were collected and assessed for the expression of muscle markers by western blot analysis. **b** Lysates were combined from several plates for immunoblotting detection of Smad7. Actin was used as loading control. **c** Co-localization of Smad7 and β-catenin was analyzed by immunofluorescence in ectopically expressing EYFP-Smad7-NLS and mcherry- β-catenin C2C12 cells. **d** Cytoplasmic and nuclear extraction was done to determine the endogenous localization of Smad7 and β-catenin in C2C12 cells. Extracellular signal-regulated kinase was utilized as cytoplasmic control and c-Jun as nuclear control. **e** Co-immunoprecipitation (Co-IP) assays were performed using Smad7 antibody to detect an interaction between endogenous β-catenin and Smad7 in extracts from mouse tibialis anterior (TA) muscle and C2C12 myoblasts. **f** Alternatively, in another separate experiment, C2C12 myoblasts were transiently transfected with Smad7-Flag. Smad7-Flag lysates were utilized for Co-IP with β-catenin or Flag antibody to detect interaction with β-catenin. IP with IgG served as controls

A time course analysis of β-catenin and myogenic markers during C2C12 differentiation indicated that β-catenin expression is enhanced along with myosin heavy chain (MyHC), muscle creatine kinase (CKM), and myogenin levels during differentiation (Fig. 1a). Endogenous Smad7 protein levels increased during differentiation as compared to myoblasts in growth conditions (Fig. 1b). Immunofluorescence analysis of Smad7- and β-catenin-expressing C2C12 cells indicates that both Smad7 and β-catenin are localized in the nucleus; interestingly, two different patterns of localization were observed. β-Catenin exhibited a diffuse pattern of localization within the nucleus in some cells while in others a very defined co-localization occurred in nuclear puncta (Fig. 1c). A cytoplasmic and nuclear fractionation demonstrated that Smad7 and β-catenin are abundant in both fractions (Fig. 1d). Based on a previous report indicating a PPI between β-catenin and Smad7 in human prostate cancer cells (PC-3U)^32^, we assessed whether Smad7 and β-catenin form a complex under these cellular conditions in cultured muscle cells and muscle tissue (mouse tibialis anterior (TA)). Total protein lysates from mouse TA and C2C12 muscle cells were subjected to Co-IP with β-catenin and Smad7 antibodies. Interestingly, Smad7 and β-catenin were precipitated together in muscle tissue (TA) and in muscle cells (Fig. 1e). The interaction was further confirmed by ectopically expressing Smad7-Flag followed by detection of β-catenin in the Co-IP (Fig. 1f).

### Smad7 physically interacts with β-catenin

**Fig. 2:**
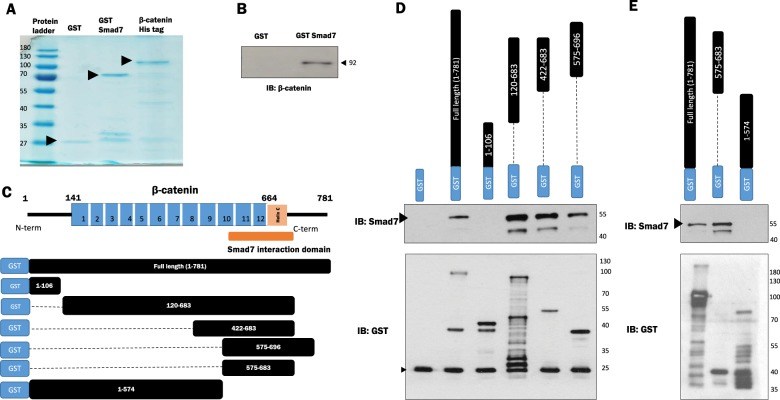
β-catenin C terminal domain (aa575–683) comprises a Smad7 interaction domain (SID). **a** Coomassie blue staining of the purified proteins GST (26 kDa), GST-Smad7 (72 kDa), and β-catenin His tag (98 kDa). **b** Glutathione-S-transferase (GST) pull down assays were performed with purified GST-Smad7 and 6× Histidine (6×His)-β-catenin fusion protein expressed in the bacteria. **c** The β-catenin protein (781aa) consists of a central region (aa 141–664) made up of 12 Armadillo repeats that are flanked by distinct N-and C-terminal domains, respectively. A specific conserved helix (Helix-C) is located proximally to the C-terminal domain, adjacent to the last ARM repeat. **d**, **e** Interaction between Smad7 and β-catenin was assessed by GST pull down assay. Purified Smad7 was incubated with GST or GST-β-catenin fusion protein fragments along with glutathione-agarose beads; detection of Smad7 protein complexed with GST-β-catenin fragments were analyzed by immunoblotting with anti-Smad7 polyclonal antibody

As the Co-IP analysis indicated Smad7 and β-catenin were in the same complex, we next clarified whether this was a direct interaction by utilizing GST pull down assays with bacterially expressed purified proteins. GST-Smad7 and 6xHistidine (6xHis)-β-catenin fusion protein were produced to conduct the assay. A Coomassie stained blot confirmed the purification of the fusion proteins (Fig. 2a). β-catenin immunoprecipitated with GST-Smad7 but not with GST-conjugated beads indicated that this interaction was direct (Fig. 2b). Next, we determined the Smad7 interaction domain (SID) on β-catenin. The 781 aa sequence of β-catenin gives rise to a structure consisting of several characteristic repeats, termed armadillo repeats, each approximately 40 aa’s long. N-terminal (NTD) and C-terminal (CTD) domains flank either end of the armadillo repeats. Helix-C indicates a conserved helix located adjacent to the last ARM repeat proximal to the CTD^35^ (Fig 2c). GST-β-catenin protein fragments corresponding to aa’s 1–106, 120–683, 422–683, 575–696, and the FL were utilized for determining the interacting region with Smad7. Smad7 interacted with FL β-catenin (as we previously identified) and β-catenin aas 120–683, 422–683 and 575–696. Smad7 did not interact with β-catenin aa1–106 therefore indicating that the region between aa575 and aa683 (Fig. 2d) is required for this interaction. To further refine this, we used additional GST-β-catenin fragments, GST-β-catenin aa575–683 and GST-β-catenin aa1–574 and conducted the interaction assay. In agreement with the above results, we observed that Smad7 interacted with β-catenin aa575–683 and not with aa1–574 leading us to conclude that the SID on β-catenin lies between aa575 and aa683 (Fig. 2e). This region spans the 10th armadillo repeat of β-catenin and a partial region extending into the CTD. Previously, this CTD domain has been associated with β-catenin *trans*activation properties and has been shown to interact with MED12, TBP (TATA-binding protein), CBP (CREB-binding protein/p300), and proteins associated with chromatin regulation^35^.

### Smad7 and β-catenin are enriched on the muscle-specific *ckm* promoter proximal region

**Fig. 3:**
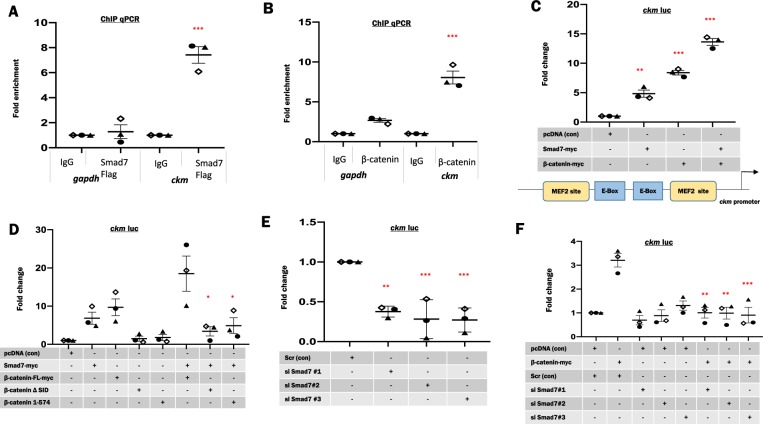
Smad7 and β-catenin enhance *ckm* proximal promoter activity. **a**, **b** For chromatin immunoprecipitation (IP), myoblasts expressing Smad7-Flag or un-transfected myoblasts were crosslinked with 1% formaldehyde followed by sonication to shear DNA (Smad7 or β-catenin). IP with anti-Flag or β-catenin antibody was carried out to precipitate protein–DNA complexes. Comparable IP with IgG antibody served as control. Recovered DNA was reverse crosslinked, purified, and subjected to quantitative PCR (qPCR) using primers specific for the *ckm* promoter to determine enrichment. Primers specific to *gapdh* were used as controls. **c** Combinations of Smad7-myc and β-catenin-myc were ectopically expressed in C2C12 cells along with a *ckm* luciferase reporter gene. *Renilla* luciferase served as transfection control. C2C12 transfected with empty vector (pcDNA) and reporter genes served as controls for ectopic expression. Cells were harvested for Luciferase determination at 48 h after changing to differentiation media (DM) post-transfection. Normalized luciferase activity was compared to the control to determine fold changes. **d** C2C12 cells were transfected with Smad7-myc, β-catenin-FL(full-length)-myc, β-catenin ΔSID, and β-catenin 1-574 alone or in combination with *ckm* luciferase reporter construct. **e** Three small interfering RNAs (siRNAs) specific for Smad7 were used to deplete the endogenous Smad7 levels in C2C12. Unprogrammed Scrambled siRNA was used as controls. **f** β-Catenin was transfected along with *ckm* luciferase. Empty vector (pcDNA) was used as a control for ectopic expression*. Renilla* was used as a control reporter to monitor transfection efficiency. Lysates were collected at 48 h after changing to DM post-transfection. The firefly luciferase activity under each condition was measured independently and normalized to *Renilla* luciferase values. Each condition is compared to the control for the three individually transfected samples to determine fold change. Each dot represents one biological replicate, which corresponds to the mean of three technical replicates. *N* = 3 biological replicates per condition. The error bars represent standard error of the mean (SEM). Dunnett’s multiple comparisons test in one-way analysis of variance using GraphPad Prism 8.0 was utilized to test for statistical significance. *p ≤ 0.05, **p ≤ 0.01, ***p ≤ 0.001

**Fig. 4:**
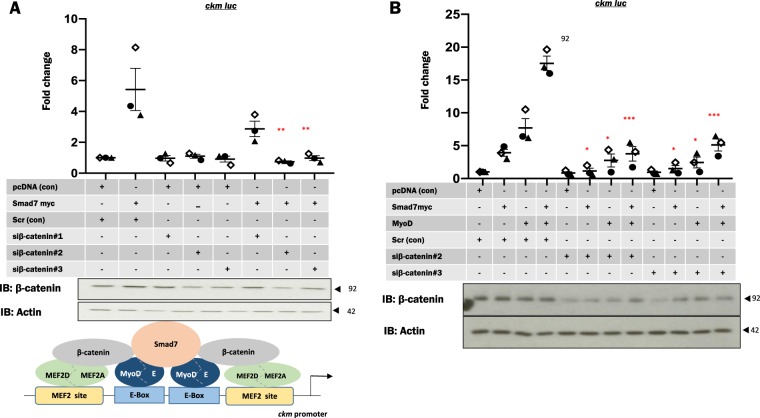
Smad7 and β-catenin co-operativity on *ckm* was mediated through MyoD. **a** C2C12 cells were transfected with small interfering RNAs targeting β-catenin to deplete the endogenous β-catenin levels. Smad7-myc was transfected along with *ckm* luciferase reporter gene. **b** MyoD and Smad7-myc alone or in combination were transfected along with *ckm* luciferase promoter activity reporter gene in depleted β-catenin condition. Empty vector (pcDNA) was used as a control for ectopic expression; *Renilla* Luciferase was used to normalize for transfection efficiency. Lysates were collected at 48 h after changing to differentiation media (DM) post-transfection. Each condition is compared to the control for the three individually transfected samples to determine fold change. Each dot represents one biological replicate, which corresponds to the mean of three technical replicates. *N* = 3 biological replicates per condition. The error bars represent standard error of the mean (SEM). Dunnett’s multiple comparisons test in one-way analysis of variance using GraphPad Prism 8.0 was utilized to test for statistical significance. *p ≤ 0.05, **p ≤ 0.01, ***p ≤ 0.001

The regulatory region of the *ckm* gene (*ckm*) has been extensively characterized and has served as a paradigm for tissue-restricted transcriptional control during myogenesis^17,36^. Our laboratory previously documented that Smad7 associates with the promoter proximal elements of *ckm*^29^. We therefore used this as a test promoter to assess the function of the β-catenin:Smad7 interaction and to interrogate the role of this complex during myogenic differentiation. First, ChIP coupled with qPCR for *ckm* and *gapdh* (control) was utilized to assess whether Smad7 and β-catenin are enriched on the *ckm* promoter proximal region. In the absence of a Smad7 antibody efficacious for ChIP, Smad7 enrichment was confirmed by Flag-Smad7 recruitment to the *ckm* promoter (Fig. 3a). Additionally, endogenous β-catenin is enriched at the *ckm* promoter in myogenic cells (Fig. 3b). Next, we utilized reporter gene assays in which Smad7 and β-catenin were exogenously expressed in C2C12 cells along with a *ckm* (−1082 to −1262) promoter fragment driving a firefly luciferase reporter gene. These data indicate that Smad7 and β-catenin *trans*activate this promoter region both alone and in combination (Fig. 3c). Based on the interaction mapping, a mammalian expression vector for β-catenin without the SID (deletion of aa575–683) was constructed. Initially, we performed reporter gene assays of the activity of these β-catenin constructs using the TOP flash luciferase reporter gene, which acts both as a β-catenin reporter and a general Wnt signaling pathway reporter as it contains 7 TCF/LEF consensus sites in which TCF/LEF proteins bind to but cannot activate the reporter gene without β-catenin. Our data indicated that the FL β-catenin activated TOP flash, whereas β-catenin 1–574 and β-catenin ΔSID could minimally activate TOP flash compared to the FL β-catenin (supplementary Fig. S1). Further, when β-catenin was ectopically expressed with Smad7, there was an enhancement in TOP flash promoter activity, whereas there was no change in TOP flash activity in conditions where Smad7 was expressed in combination with β-catenin 1–574 and β-catenin ΔSID. These data revealed that, while Smad7 was able to enhance the function of the intact β-catenin, it did not co-operate with β-catenin 1–574 and β-catenin ΔSID in reporter gene assays (supplementary Fig. S1). We next deleted the SID domain to test whether it might prevent the cooperative *trans*activation mediated by Smad7 and β-catenin. To test this idea, Smad7, FL β-catenin (β-catenin-FL), β-catenin (aa1–574, ΔSID) along with a *ckm* luciferase construct were ectopically expressed in C2C12 cells alone or in combination. These data indicate that, while Smad7 and the β-catenin-FL enhanced the *ckm* promoter, this activity was substantially reduced in cells expressing Smad7 in combination with β-catenin aa1–574 or β-catenin ΔSID (Fig. 3d). These data support the conclusion that the β-catenin SID is crucial for the cooperative interaction of Smad7 and β-catenin on the *ckm* promoter. To address this cooperativity in a different way, we next investigated the effect of perturbation in Smad7 expression on the function of β-catenin on the *ckm* promoter using siRNA technology. Endogenous Smad7 levels were depleted using three independent siRNAs (supplementary Fig. S2) and reporter gene analysis indicated a pronounced decrease in *ckm* promoter activity when compared to the control (Fig. 3e). Ectopic expression of β-catenin in Smad7-depleted cells exhibited a reduction in *ckm* promoter induction consistent with the interpretation that β-catenin requires Smad7 in order to affect promoter activation (Fig. 3f). To further test this idea, we correspondingly depleted β-catenin expression using three independent siRNAs. Western blot analysis verified a considerable reduction in protein levels of β-catenin using two different siRNAs (si#2 and #3) as compared to controls. Under conditions in which endogenous β-catenin was depleted, we observed that Smad7 mediated *ckm* promoter activation was markedly reduced (Fig. 4a). These data indicate that both Smad7 and β-catenin cooperatively activate the *ckm* promoter. Previously, it was shown that MyoD, the archetypal MRF^37^, interacts with β-catenin^38^. To test this hypothesis, endogenous β-catenin levels were depleted in C2C12 using two different siRNAs. MyoD and Smad7-myc either alone or in combination were then transfected along with the *ckm* reporter gene in β-catenin-depleted cells. This analysis revealed that *ckm* promoter activity increased by Smad7 or/and MyoD was reduced by depletion of β-catenin (Fig. 4b). These results further support the conclusion that β-catenin enhances Smad7- and MyoD-driven potentiation of *ckm* promoter activity. These observations in combination with previously reported data indicate that the Smad7:β-catenin interaction may be tethered by MyoD and is required for full *ckm* promoter activation.

### The minimal SID on β-catenin functions as a *trans*dominant inhibitor of β-catenin activity

**Fig. 5:**
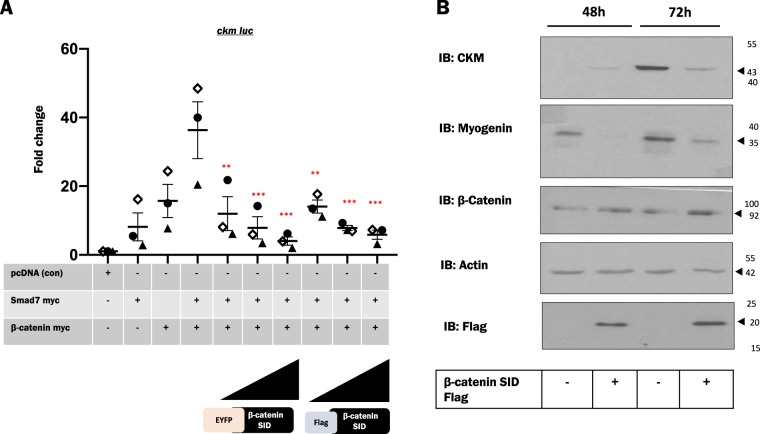
β-Catenin aa575-683 (Smad7- interacting domain (SID)) functions as a dominant-negative inhibitor of endogenous β-catenin activity. **a** Smad7-myc and β-catenin-myc alone or in combination were transfected along with increasing amounts of either EYFP-β-catenin SID or Flag β-catenin SID and *ckm* luciferase construct. Empty vector (pcDNA) was used as a control. Lysates were collected at 48 h after changing to differentiation media (DM) post-transfection. Each condition was compared to the control for the three individually transfected samples to determine fold change. Each dot represents one biological replicate, which corresponds to the mean of three technical replicates. *N* = 3 biological replicates per condition. The error bars represent standard error of the mean (SEM). Dunnett’s multiple comparisons test in one-way analysis of variance using GraphPad Prism 8.0 was utilized to test for statistical significance. **p ≤ 0.01, ***p ≤ 0.001. **b** Total cell lysates of ectopically expressing Flag β-catenin SID were collected at 48 and 72 h after changing to DM post-transfection. Un-transfected cells served as controls. Cell lysates were analyzed for endogenous β-catenin, muscle markers (Ckm, Myogenin), and Flag (β-catenin SID) by western blot analysis. An actin blot indicated protein loading of samples

Since our data suggested that the SID is crucial for the function of Smad7 and β-catenin on the *ckm* promoter, we next considered the possibility that this domain of β-catenin might function as a more general *trans*dominant repressor (dominant negative) of endogenous β-catenin activity. To test this idea, we constructed a mammalian expression plasmid for tagged β-catenin aa575–683. Smad7 and β-catenin were ectopically expressed in C2C12 cells along with increasing concentrations of either EYFP-β-catenin aa575–683 (EYFP-SID) or Flag β-catenin aa575–683 (Flag-SID). Reporter gene analysis revealed that *ckm* promoter activity was significantly reduced in Smad7- and β-catenin-expressing cells in the presence of either EYFP-SID or Flag-SID (Fig 5a). These data indicate that the β-catenin SID can function as a dominant-negative inhibitor of β-catenin function. A similar analysis using a *sprr1a* promoter luciferase reporter gene that is not regulated by MyoD (data not shown) was unaffected by SID expression, suggesting that the effect of SID on *ckm* is specific (supplementary Fig. S3). We subsequently assessed the effect of the SID domain on myogenesis by analyzing the protein levels of myogenic markers with and without SID expression. Immunoblot analysis from ectopically expressed SID in C2C12 cells exhibited reduced protein levels of CKM and Myogenin as compared to controls indicating an overall repression of myogenesis by exogenous SID expression (Fig. 5b). Collectively these data suggested that the β-catenin SID can inhibit the activity of endogenous β-catenin and the myogenic differentiation program.

### Smad7:β-catenin complex interact with the Mediator kinase complex subunits (MED13 and 12)

**Fig. 6:**
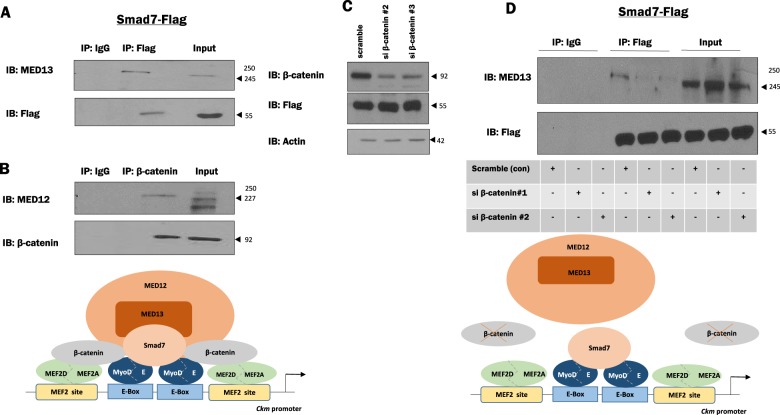
Mediator subunit 13 (MED13) associates with Smad7 in a β-catenin-dependent manner. **a** C2C12 cells were transiently transfected with Smad7-flag. Lysates were collected at 24 h after replenishing media post-transfection. Co-immunoprecipitation (Co-IP) assays were performed using anti-Flag antibody to test an interaction between endogenous MED13 and Smad7. **b** Co-IP assays were performed using β-catenin antibody to test for an interaction between endogenous MED12 and β-catenin. IP with IgG served as controls. **c** Small interfering RNAs (siRNAs) specific for β-catenin were used to deplete the endogenous β-catenin levels in C2C12, Scrambled siRNA served as controls. Lysates were collected at 48 h post-transfection and immunoblotted for β-catenin, anti-Flag (for Smad7-Flag), and Actin (loading control). **d** Co-IP with anti-Flag was repeated in depleted β-catenin conditions to assess the interaction between endogenous MED13 and Smad7

Previous studies^39^ and our unpublished observations confirm that β-catenin directly interacts with MED12. Since it is established that MED12 and MED13 form an integral part of the mediator kinase module^39,40^, we hypothesized that the composite function of the Smad7:β-catenin interaction might be to recruit the MED kinase module. This idea is supported by our Co-IP analysis in which we observed that Smad7 and MED13 are in the same precipitated protein complex (Fig. 6a). Furthermore, we also validated the previously reported MED12:β-catenin interaction (Fig. 6b). Finally, we observed that the Smad7:MED13 interaction was disrupted when endogenous β-catenin was depleted (Fig. 6c, d). The Smad7:MED13 interaction thus constitutes a novel observation in characterizing Smad7 as a component of the transcriptional machinery-linking promoter activity to the Mediator kinase complex.

## Discussion

### Smad7:β-catenin as an essential component of muscle enhanceosomes

**Fig. 7:**
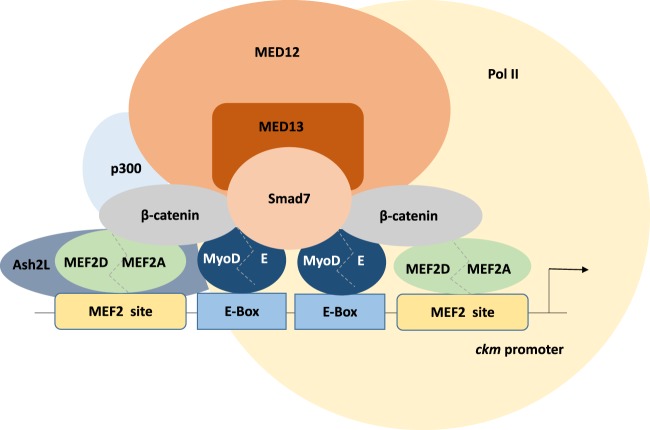
Proposed model of Smad7:β-catenin integration into the transcriptional holocomplex on the *ckm* proximal promoter region. The core *ckm* promoter proximal region contains essential binding sites for MEF2 proteins and MyoD/E protein complexes. β-catenin has also been previously implicated in binding to MyoD and MEF2 transcription factors. The trithorax group protein Ash2L was previously established as one of the interacting partners of MEF2D/2A. Based on the published work and the Smad7:β-catenin:MED12/13 contribution identified herein, an integrated model of the transcriptional regulation of the *ckm* gene is proposed

Despite a considerable body of literature implicating Wnt-β-catenin signaling in muscle differentiation^26,41–43^, there is a surprising lack of mechanistic insight into how it fulfills this role. Moreover, recent in vitro and in vivo data implicating Smad7 in the control of muscle gene expression is also not understood mechanistically. Interestingly, in one study^44^ a correlation between TGF-β and Wnt signaling was investigated where TGF-β1 was observed to control the differentiation of fibroblasts to myofibroblasts by upregulating Wnt signaling. Here we provide evidence of a direct functional interaction between Smad7 and β-catenin that serves a fundamental role in recruiting Mediator components to the well-characterized *ckm* gene. Our data indicate that β-catenin is inefficiently recruited to the *ckm* gene enhancer in muscle cells when Smad7 is depleted and corresponding loss of β-catenin recruitment renders this gene incapable of responding to essential differentiation cues (Fig. 7). Here we present evidence indicating that a Smad7:β-catenin complex is a critical part of the transcriptional machinery at a key muscle promoter proximal element, being intrinsically necessary as a co-regulator for the MRFs in the myogenic gene expression program.

### Mediator recruitment to *ckm* by β-catenin/Smad7

The Mediator complex was initially characterized in budding yeast^45^ and has since been established as fulfilling an essential function in RNA polymerase II-mediated gene transcription from flies to mammals^46^. The multi-subunit compositional complexity of the Mediator holocomplex has proved a substantial challenge to define the full extent of its properties but what is apparent is that its fundamental role is to provide a functional bridge between transcriptional regulatory proteins bound to gene enhancers and the general transcription machinery assembled at core promoters^47–53^. The interaction of different Mediator subunits with a variety of transcription factors thus allows a myriad of cellular signaling events that converge on the transcription factors to be subsequently relayed to the transcriptional machinery and ultimately programs of gene transcription^54^. Thus, this essential activity in promoting signal-dependent transcriptional pre-initiation complex (PIC) assembly and stabilization renders many Mediator subunits essential for life, since gene targeting in mice has revealed that many subunits prove embryonic lethal when deleted and in yeast all Pol II-regulated genes are dependent on Mediator^52,55–57^. The experimental dissection of Mediator has been aided by the characterization of four relatively stable sub-complexes that have been designated as the head, middle, tail, and kinase modules. In our study, we have documented that the Smad7:β-catenin complex specifically associates with the MED12 and 13 subunits of the Mediator kinase module. The kinase domain is speculated to fulfill a transient regulatory function in Mediator by promoting recruitment of all four Mediator domains to enhancers which then transitions to a core promoter-bound Mediator complex in which the kinase module is absent. We therefore propose that Mediator recruitment to the *ckm* enhancer is mediated by the Smad7:β-catenin complex in muscle cells (Fig. 7).

### Implications of β-catenin:Smad7 interaction in Rhabdomyosarcoma (RMS)

In view of the role played by β-catenin: Smad7, it is perhaps worth consideration of the implications of this in the context of the pathology of RMS, a soft tissue pediatric cancer with features of muscle^58^. Previously, we reported that constitutive glycogen synthase kinase 3 (GSK3) activity is a feature of the embryonal form of RMS (there are two general categories: alveolar (ARMS) and embryonal (ERMS)). The result of this GSK3 activity is perpetual proteosomal degradation of β-catenin since this is the primary function of the APC-GSK3 complex in canonical Wnt signaling^59^. Upon Wnt signaling stimulation, GSK3 activity is substantially reduced and proteosomal degradation of β-catenin ceases, resulting in its cytoplasmic accumulation and translocation to the nucleus where it activates transcription in combination with other transcriptional regulators, such as TCF/LEF. Based on these previous observations and the current study, it is worth speculating that the absence of nuclear β-catenin function due to constitutive GSK3 activity contributes to the differentiation defect in ERMS. Promoting terminal differentiation of the myo-like cells in RMS is seen as a potentially effective therapeutic target, since differentiation, by nature, results in cell cycle withdrawal and cessation of proliferation which would cause tumor regression. There are several lines of indirect evidence supporting this idea; we recently reported that Myogenin is expressed at high levels in RMS but, despite the role of this MRF as a key terminal effector of the myogenic differentiation program, it is functionally inactive in these cells. It is therefore possible that the lack of nuclear β-catenin underlies this functional impairment due to the failure to recruit Mediator to connect Myogenin function to PIC assembly in these ERMS cells. In support of this possibility, we observed that Smad7:β-catenin is tethered to muscle promoters by its interaction with MRFs (such as MyoD and Myogenin) bound to E boxes on muscle promoter/enhancer regions. Another line of circumstantial evidence supporting this notion is that pharmacological GSK3 inhibitors have been reported to force RMS cells into a more differentiated cellular phenotype, suggesting that re-constitution of β-catenin function in RMS cells promotes their differentiation and could potentially be antitumorigenic. Further clarification of the role of β-catenin:Smad7 as a therapeutic target in RMS is therefore warranted.

### Further implications of p38 mitogen-activated protein kinase (MAPK) signaling on *ckm* regulation

Phospho-dependent protein interactions are a signature of β-catenin function. There is ample evidence of kinase-mediated phosphorylation modulating the affinity of PPIs in the canonical Wnt pathway, resulting in important outcomes for Wnt-dependent target gene activation^33^. Specifically, we recently reported that a p38 MAPK-dependent interaction with the MEF2 transcription factor enhances β-catenin nuclear retention and activity^33^. During differentiation, MyoD and MEF2 bind muscle-specific promoters and enhancers, leading to the recruitment of co-activators (including p300) and the basal transcriptional machinery to establish a transcriptionally poised promoter. The previously implicated mechanism is that p38 MAPK activates *ckm* expression by phosphorylation-dependent recruitment of the histone methyltransferase Ash2L by MEF2D^60^. In view of the current observations, it is also possible that p38 MAPK may promote β-catenin recruitment to *ckm* through its interaction with MEF2.

In summary, we have documented a PPI between Smad7 and β-catenin that may serve a fundamental role in the control of myogenic gene transcription. These observations may have implications for our understanding of the molecular control of myogenic differentiation during embryonic development and adult muscle regeneration.

## Supplementary information

Supplementary data

Supplementary figures

## References

[1] Mok GF, Sweetman D (2011). Many routes to the same destination: lessons from skeletal muscle development. Reproduction.

[2] Musumeci G (2015). Somitogenesis: from somite to skeletal muscle. Acta Histochem..

[3] Chal J, Pourquie O (2017). Making muscle: skeletal myogenesis in vivo and in vitro. Development.

[4] Toto PC, Puri PL, Albini S (2016). SWI/SNF-directed stem cell lineage specification: dynamic composition regulates specific stages of skeletal myogenesis. Cell. Mol. Life Sci..

[5] Wu Z (2000). p38 and extracellular signal-regulated kinases regulate the myogenic program at multiple steps. Mol. Cell. Biol..

[6] Pownall ME, Gustafsson MK, Emerson CP (2002). Myogenic regulatory factors and the specification of muscle progenitors in vertebrate embryos. Annu. Rev. Cell Dev. Biol..

[7] Chan CY (2011). Identification of differentially regulated secretome components during skeletal myogenesis. Mol. Cell. Proteomics.

[8] Garcia-Prat L, Sousa-Victor P, Munoz-Canoves P (2017). Proteostatic and metabolic control of stemness. Cell Stem Cell.

[9] Mauro A (1961). Satellite cell of skeletal muscle fibers. J. Biophys. Biochem. Cytol..

[10] Sincennes MC, Brun CE, Rudnicki MA (2016). Concise review: Epigenetic regulation of myogenesis in health and disease. Stem Cells Transl. Med..

[11] Black BL, Olson EN (1998). Transcriptional control of muscle development by myocyte enhancer factor-2 (MEF2) proteins. Annu. Rev. Cell Dev. Biol..

[12] Imbriano, C. & Molinari, S. Alternative splicing of transcription factors genes in muscle physiology and pathology. *Genes (Basel)* **9**, 10.3390/genes9020107 (2018). 10.3390/genes9020107 PMC5852603 29463057

[13] Ferri P (2009). Expression and subcellular localization of myogenic regulatory factors during the differentiation of skeletal muscle C2C12 myoblasts. J. Cell. Biochem..

[14] Lu J, McKinsey TA, Zhang CL, Olson EN (2000). Regulation of skeletal myogenesis by association of the MEF2 transcription factor with class II histone deacetylases. Mol. Cell.

[15] Edmondson DG, Lyons GE, Martin JF, Olson EN (1994). Mef2 gene expression marks the cardiac and skeletal muscle lineages during mouse embryogenesis. Development.

[16] Ornatsky OI, Andreucci JJ, McDermott JC (1997). A dominant-negative form of transcription factor MEF2 inhibits myogenesis. J. Biol. Chem..

[17] Johnson JE, Wold BJ, Hauschka SD (1989). Muscle creatine kinase sequence elements regulating skeletal and cardiac muscle expression in transgenic mice. Mol. Cell. Biol..

[18] McCord RP, Zhou VW, Yuh T, Bulyk ML (2011). Distant cis-regulatory elements in human skeletal muscle differentiation. Genomics.

[19] Fujisawa-Sehara A (1992). Differential trans-activation of muscle-specific regulatory elements including the mysosin light chain box by chicken MyoD, myogenin, and MRF4. J. Biol. Chem..

[20] Estrella NL (2015). MEF2 transcription factors regulate distinct gene programs in mammalian skeletal muscle differentiation. J. Biol. Chem..

[21] Grifone R (2005). Six1 and Six4 homeoproteins are required for Pax3 and Mrf expression during myogenesis in the mouse embryo. Development.

[22] Laclef C (2003). Altered myogenesis in Six1-deficient mice. Development.

[23] Liu Y, Chu A, Chakroun I, Islam U, Blais A (2010). Cooperation between myogenic regulatory factors and SIX family transcription factors is important for myoblast differentiation. Nucleic Acids Res..

[24] Tobin SW (2016). Regulation of Hspb7 by MEF2 and AP-1: implications for Hspb7 in muscle atrophy. J. Cell. Sci..

[25] Alli NS (2013). Signal-dependent fra-2 regulation in skeletal muscle reserve and satellite cells. Cell Death Dis..

[26] Suzuki A, Pelikan RC, Iwata J (2015). WNT/beta-catenin signaling regulates multiple steps of myogenesis by regulating step-specific targets. Mol. Cell. Biol..

[27] Suzuki A, Scruggs A, Iwata J (2015). The temporal specific role of WNT/beta-catenin signaling during myogenesis. J. Nat. Sci..

[28] Otto A (2008). Canonical Wnt signalling induces satellite-cell proliferation during adult skeletal muscle regeneration. J. Cell. Sci..

[29] Kollias HD, Perry RL, Miyake T, Aziz A, McDermott JC (2006). Smad7 promotes and enhances skeletal muscle differentiation. Mol. Cell. Biol..

[30] Cohen TV, Kollias HD, Liu N, Ward CW, Wagner KR (2015). Genetic disruption of Smad7 impairs skeletal muscle growth and regeneration. J. Physiol..

[31] Miyake T, Alli NS, McDermott JC (2010). Nuclear function of Smad7 promotes myogenesis. Mol. Cell. Biol..

[32] Edlund S (2005). Interaction between Smad7 and beta-catenin: importance for transforming growth factor beta-induced apoptosis. Mol. Cell. Biol..

[33] Ehyai S (2016). A p38 mitogen-activated protein kinase-regulated myocyte enhancer factor 2-beta-catenin interaction enhances canonical Wnt signaling. Mol. Cell. Biol..

[34] Ehyai, S. et al. FMRP recruitment of beta-catenin to the translation pre-initiation complex represses translation. *EMBO Rep*. **19**, 10.15252/embr.201745536 (2018). 10.15252/embr.201745536 PMC6280795 30361391

[35] Valenta T, Hausmann G, Basler K (2012). The many faces and functions of beta-catenin. EMBO J..

[36] Chamberlain JS, Jaynes JB, Hauschka SD (1985). Regulation of creatine kinase induction in differentiating mouse myoblasts. Mol. Cell. Biol..

[37] Sartorelli V, Puri PL (2018). Shaping gene expression by landscaping chromatin architecture: lessons from a master. Mol. Cell.

[38] Kim CH, Neiswender H, Baik EJ, Xiong WC, Mei L (2008). Beta-catenin interacts with MyoD and regulates its transcription activity. Mol. Cell. Biol..

[39] Kim S, Xu X, Hecht A, Boyer TG (2006). Mediator is a transducer of Wnt/beta-catenin signaling. J. Biol. Chem..

[40] Furumoto T (2007). A kinase subunit of the human mediator complex, CDK8, positively regulates transcriptional activation. Genes Cells.

[41] Tsivitse S (2010). Notch and Wnt signaling, physiological stimuli and postnatal myogenesis. Int. J. Biol. Sci..

[42] Cisternas P, Henriquez JP, Brandan E, Inestrosa NC (2014). Wnt signaling in skeletal muscle dynamics: myogenesis, neuromuscular synapse and fibrosis. Mol. Neurobiol..

[43] Snyder CM (2013). MEF2A regulates the Gtl2-Dio3 microRNA mega-cluster to modulate WNT signaling in skeletal muscle regeneration. Development.

[44] Vallee A, Lecarpentier Y, Guillevin R, Vallee JN (2017). Interactions between TGF-beta1, canonical WNT/beta-catenin pathway and PPAR gamma in radiation-induced fibrosis. Oncotarget.

[45] Kelleher RJ, Flanagan PM, Kornberg RD (1990). A novel mediator between activator proteins and the RNA polymerase II transcription apparatus. Cell.

[46] Kornberg RD (2005). Mediator and the mechanism of transcriptional activation. Trends Biochem. Sci..

[47] Malik S, Roeder RG (2005). Dynamic regulation of pol II transcription by the mammalian Mediator complex. Trends Biochem. Sci..

[48] Kim YJ, Bjorklund S, Li Y, Sayre MH, Kornberg RD (1994). A multiprotein mediator of transcriptional activation and its interaction with the C-terminal repeat domain of RNA polymerase II. Cell.

[49] Baek HJ, Kang YK, Roeder RG (2006). Human Mediator enhances basal transcription by facilitating recruitment of transcription factor IIB during preinitiation complex assembly. J. Biol. Chem..

[50] Mittler G, Kremmer E, Timmers HT, Meisterernst M (2001). Novel critical role of a human Mediator complex for basal RNA polymerase II transcription. EMBO Rep..

[51] Taatjes DJ (2010). The human Mediator complex: a versatile, genome-wide regulator of transcription. Trends Biochem. Sci..

[52] Soutourina J, Wydau S, Ambroise Y, Boschiero C, Werner M (2011). Direct interaction of RNA polymerase II and mediator required for transcription in vivo. Science..

[53] Thompson CM, Young RA (1995). General requirement for RNA polymerase II holoenzymes in vivo. Proc. Natl Acad. Sci. USA.

[54] Myers LC, Kornberg RD (2000). Mediator of transcriptional regulation. Annu. Rev. Biochem..

[55] Bjorklund S, Gustafsson CM (2005). The yeast Mediator complex and its regulation. Trends Biochem. Sci..

[56] Soutourina J (2018). Transcription regulation by the Mediator complex. Nat. Rev. Mol. Cell Biol..

[57] Holstege FC (1998). Dissecting the regulatory circuitry of a eukaryotic genome. Cell..

[58] Paulino AC, Okcu MF (2008). Rhabdomyosarcoma. Curr. Probl. Cancer..

[59] Dionyssiou MG, Ehyai S, Avrutin E, Connor MK, McDermott JC (2014). Glycogen synthase kinase 3beta represses MYOGENIN function in alveolar rhabdomyosarcoma. Cell Death Dis..

[60] Rampalli S (2007). p38 MAPK signaling regulates recruitment of Ash2L-containing methyltransferase complexes to specific genes during differentiation. Nat. Struct. Mol. Biol..

