# Supplementary material for: Smad7:β-catenin complex regulates myogenic gene transcription
Source: Cell Death Dis. 2019 May 16;10(6):387. doi: 10.1038/s41419-019-1615-0 (PMC6522533; doi:10.1038/s41419-019-1615-0)
Supplement: Supplementary file 1 — Supplementary data [file 41419_2019_1615_MOESM1_ESM.pdf]

Supplementary data

Figure S1. Combinations of β-catenin-FL(full-length)-myc, β-catenin Δ SID, β-catenin 1-574, Smad7-myc were ectopically expressed in C2C12 cells along with a TOP flash luciferase reporter gene. Renilla luciferase served as transfection control. C2C12 cells transfected with empty vector (pcDNA) and reporter genes served as controls for ectopic expression. Cells were harvested for Luciferase determination at 12 h after changing to fresh growth media post transfection. Normalized luciferase activity was compared to the control to determine fold changes.

Figure S2. Three siRNAs specific for Smad7 were used to deplete the endogenous Smad7 levels in C2C12. Unprogrammed Scrambled siRNA was used as controls. Lysates were collected at 48 h post transfection and immunoblotted with the designated Smad7 antibodies from two different sources (one generated in house, abcam). Lysates were further immunoblotted for myc (Smad7-myc) and actin.

Figure S3. A previously characterized sprr1a promoter-luciferase reporter gene that is not regulated by the myogenic regulators served as a control promoter for general transcriptional effects since it lacks defined MEF2 or MyoD binding sites. Activated MEK (MEK R4F) was used as a positive control for sprr1a as previously characterized. Lysates were collected at 48 h after changing to differentiation media (DM) post-transfection. Each condition was compared to the control for the three individually transfected samples to determine fold change. Each dot represents one biological replicate, which corresponds to the mean of 3 technical replicates. N= 2 biological replicates per condition. The error bars represent standard error of the mean (SEM).
