# Supplementary figures and images for: Smad7:β-catenin complex regulates myogenic gene transcription
Source: Cell Death Dis. 2019 May 16;10(6):387. doi: 10.1038/s41419-019-1615-0 (PMC6522533; doi:10.1038/s41419-019-1615-0)

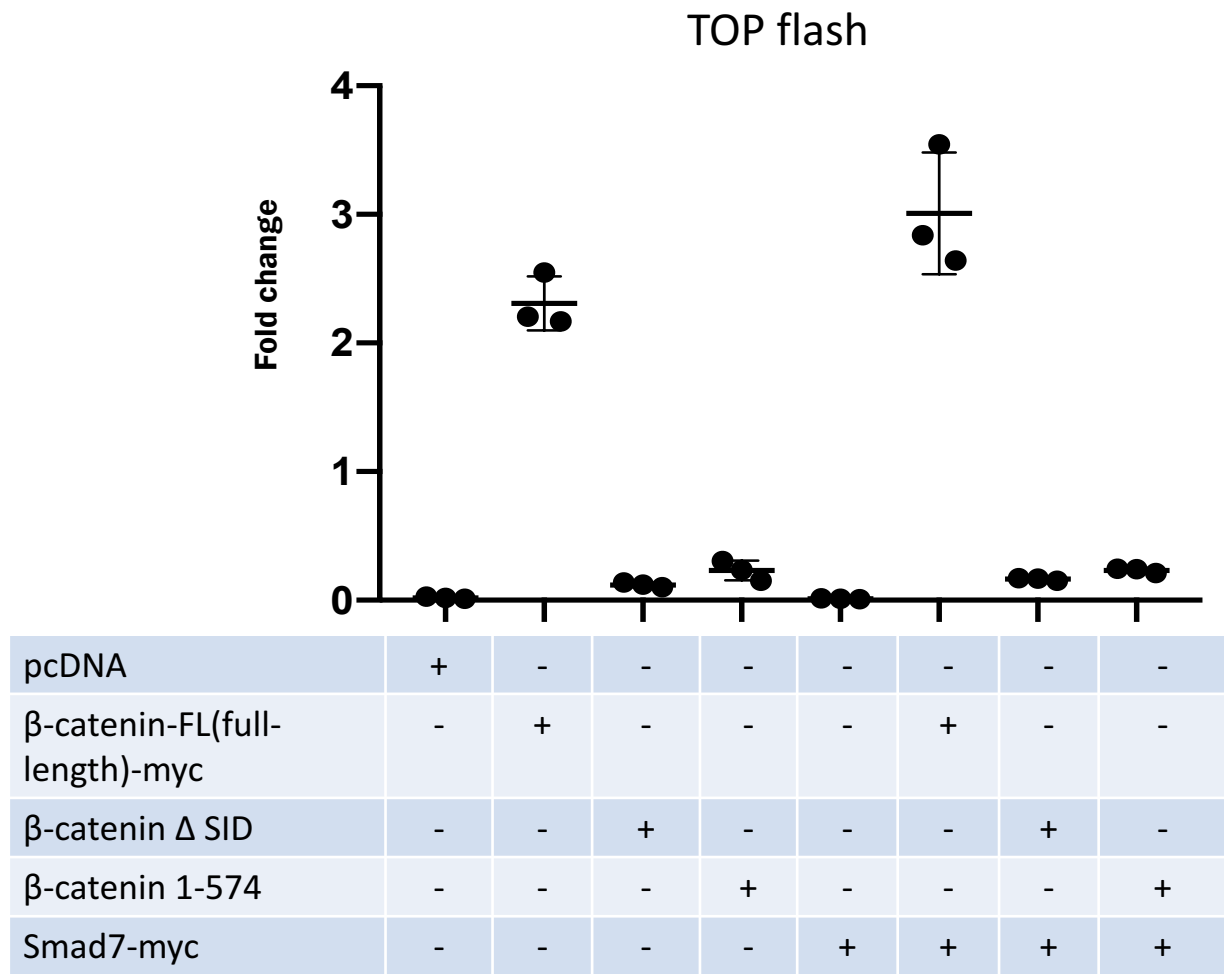

**Figure S1. TOP flash reporter assay**

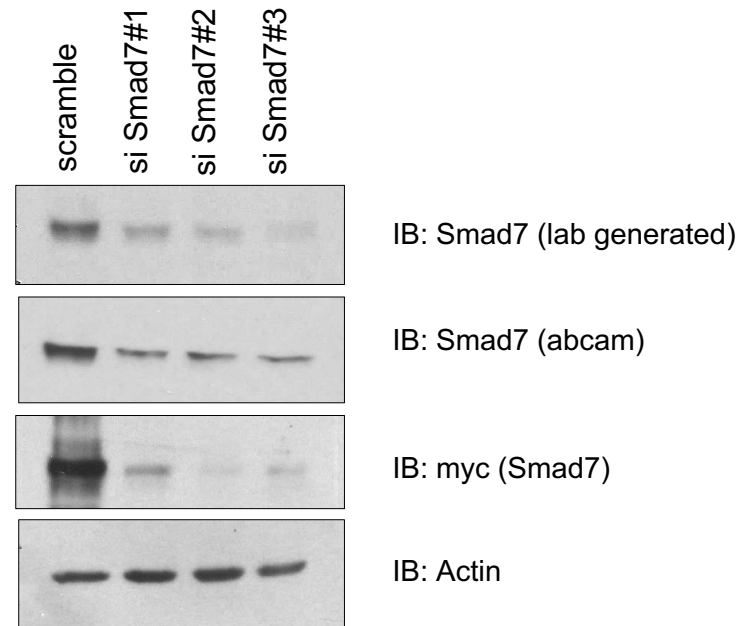

**Figure S2. Smad7 depletion by siRNA**

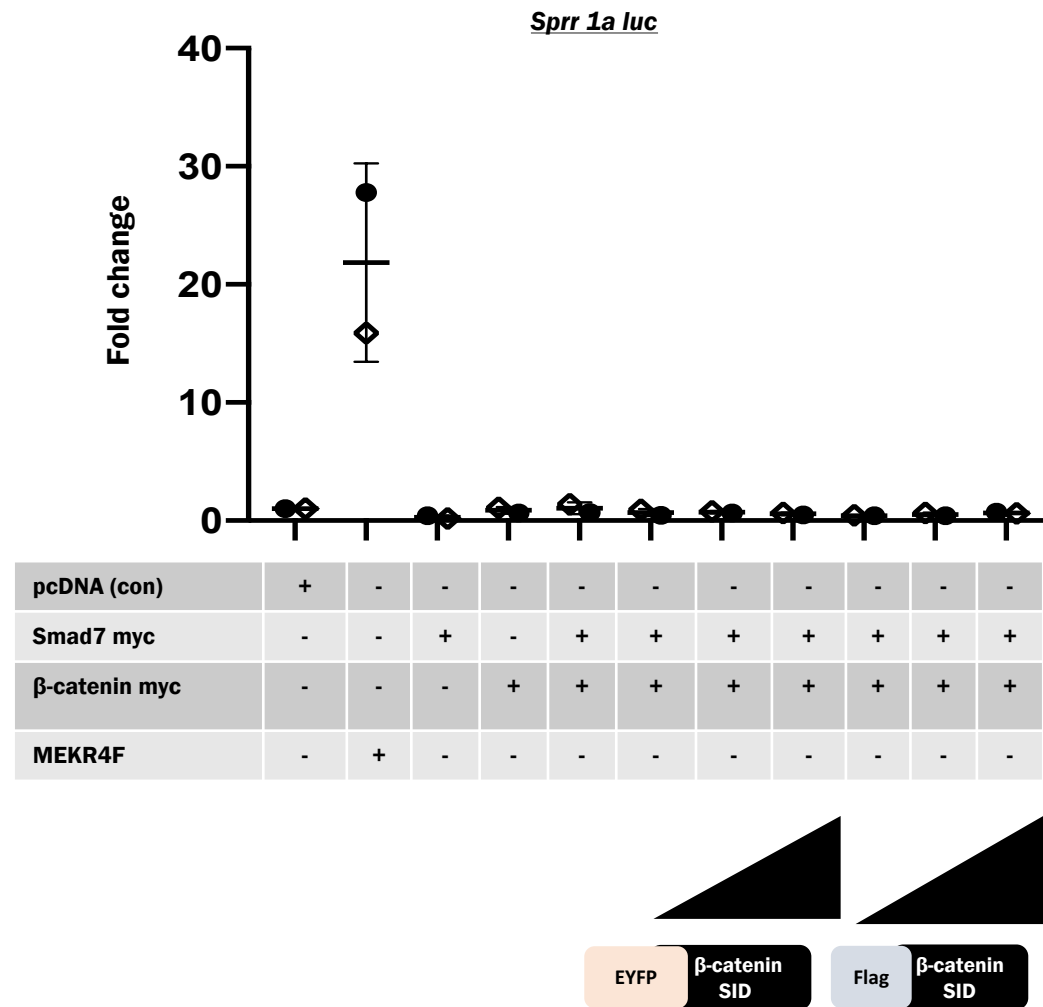

Supplement: Supplementary file 2 — Supplementary figures [file 41419_2019_1615_MOESM2_ESM.pdf]
